# Profound hypoxemia and hypotension during posterior spinal fusion in a spinal muscular atrophy child with severe scoliosis: a case report

**DOI:** 10.1186/s12871-024-02537-2

**Published:** 2024-04-18

**Authors:** Qian Shu, Yulei Dong, Weiyun Chen, Jianxiong Shen

**Affiliations:** 1 Department of Anesthesiology, Peking Union Medical College Hospital, Beijing, China; 2 Department of Orthopedic Surgery, Peking Union Medical College Hospital, Beijing, China

**Keywords:** Case report, Spinal muscular atrophy, Scoliosis, Hypoxemia, Surgical positioning

## Abstract

**Background:**

Anesthesia for spinal muscular atrophy (SMA) patients undergoing spinal deformity surgery is challenging. We report an unusual case of an SMA girl who developed severe intraoperative hypoxemia and hypotension during posterior spinal fusion related with surgical positioning.

**Case presentation:**

A 13-yr-old girl diagnosed with SMA type 2, severe kyphoscoliosis and thoracic deformity was scheduled for elective posterior spinal fusion. She developed severe hypoxemia and profound hypotension intraoperatively in the prone position with surgical table tilted 45° to the right. Though transesophageal echocardiography (TEE) could not be performed due to limited mouth opening, her preoperative computed tomography revealed a severely distorted thoracic cavity with much reduced volume of the right side. A reasonable explanation was when the surgeons performed surgical procedure with the tilted surgical table, the pressure was directly put on the shortest diameter of the significantly deformed thoracic cavity, causing severe compression of the pulmonary artery, resulting in both hypoxemia and hypotension. The patient stabilized when the surgical table was tilted back and successfully went through the surgery in the leveled prone position.

**Conclusions:**

Spinal fusion surgery is beneficial for SMA patients in preventing scoliosis progression and improving ventilation. However, severe scoliosis and thoracic deformities put them at risk of both hemodynamic and respiratory instability during surgical positioning. When advanced monitoring like TEE is not practical intraoperatively, preoperative imaging may help with differential diagnosis, and guide the surgical positioning to minimize mechanical compression of the thoracic cavity, thereby helping the patient complete the surgery safely.

## Background

Posterior spinal fusion is a common procedure to treat scoliosis that requires prone position intraoperatively to provide surgical access to the spine [1]. However, the prone position may compress the chest wall, reduce cardiac output, and cause hemodynamic changes [2, 3]. These pathophysiological changes can be more dramatic in patients with thoracic deformity and even worse in children with their more compliant chest wall [4]. We present a case of a child with spinal muscular atrophy (SMA) and severe scoliosis who experienced not only profound hypotension but severe hypoxemia simultaneously during posterior spine surgery.

## Case presentation

Informed consent for publication including identifying information/images was obtained from the patient’s legal guardian. A 13-yr-old girl with severe kyphoscoliosis was scheduled for elective posterior spinal fusion surgery. She presented symptoms of muscle weakness, difficulty in sitting and holding up the head, hyporeflexia, and growth retardation at 9 months of age. Genetic analysis showed homozygous deletion of the *SMN1* gene and 3 copies of *SMN2* gene, and she was diagnosed as SMA type 2. She developed progressive scoliosis and kyphosis at the age of eight.

**Fig. 1:**
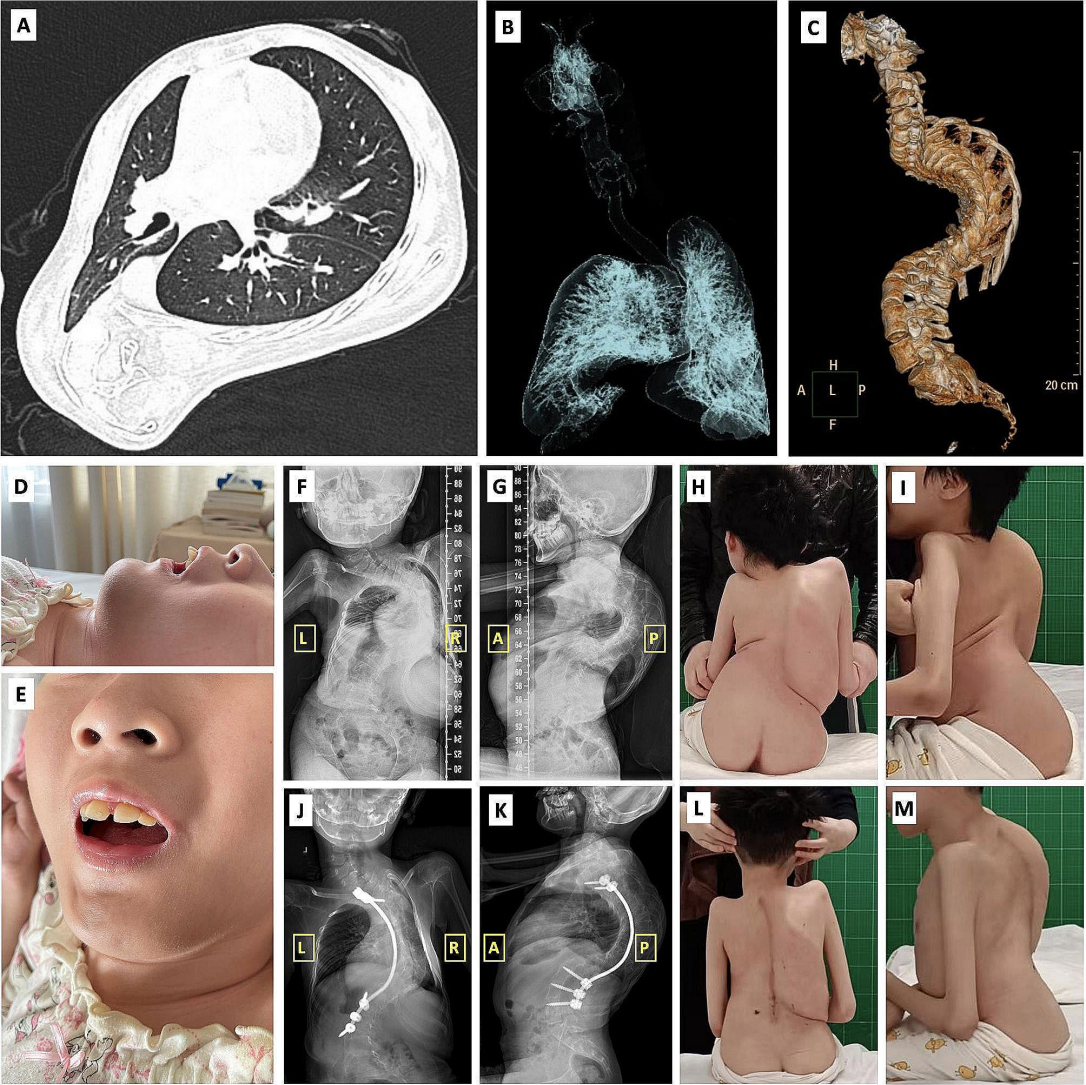
Clinical features of the patient. **A, B**) Chest CT and 3D airway tree reconstruction, showing thoracic asymmetry, bronchial stenosis, and underexpansion of the right lung. **C**) Preoperative 3D reconstruction of spine CT. **D, E**) Airway assessment. The girl is presented with very limited mouth opening for less than one finger. **F, G**) Preoperative X-ray images. **H, I**) Preoperative general observation shows severe kyphoscoliosis and multiple joint contractures. **J, K**) Postoperative X-ray images. **L, M**) Postoperative general observation shows improved kyphoscoliosis and sitting balance

The girl had no history of other surgeries and had not received general anesthesia previously. On physical examination, she had significant muscle weakness and was unable to sit without support. In addition to severe kyphoscoliosis, the girl has multiple joint contractures including flexion deformities of elbows, wrists, hips, and knees. Her preoperative magnetic resonance imaging (MRI) showed no spinal cord or other soft tissue abnormalities other than scoliosis. For preoperative evaluation, though without history of recurrent pulmonary infection, her preoperative computed tomography (CT) showed thoracic asymmetry, bronchial stenosis in the middle and inferior lobes of the right lung, and atelectasis of the inferior lobe of the right lung (Fig. 1). She had poor pulmonary function (FEV1 0.39 L, FVC 0.44 L) and had been treated with nocturnal noninvasive ventilation for two years. Also, because of difficulties in mouth opening and chewing caused by muscle atrophy and temporomandibular joint contracture, the girl had to take semisolid food and had severe dysplasia (weight 20 kg, height 100 cm). To optimize her preoperative status, multidisciplinary team was consulted. A physiatrist guided her respiratory prehabilitation preoperatively. The parameters for noninvasive ventilation were optimized by a respirologist. Her arterial blood gas (pH 7.39, PaO_2_ 81mmHg, PaCO_2_ 44mmHg) was acceptable prior to surgery. She had normal electrocardiogram and echocardiography otherwise. A nutritionist assessed her nutritional status and formulated nutritional treatment, including setting daily caloric goals with oral nutrition supplements and enhancing protein intake. Halo-gravity traction had been applied to control the progression of scoliosis for one year. For SMA treatment, she had received 6 doses of intrathecal injection of Nusinersen prior to surgery. After the above preoperative optimization, considering her kyphoscoliosis progressed rapidly despite conservative treatment (Cobb’s angle 125° for thoracic scoliosis and 122° for kyphosis preoperatively, Fig. 1), posterior spinal fusion was decided to be performed.

For anesthesia management, as the girl presented several indicators for an extremely difficult airway, including Mallampati class IV, significantly recessed mandible, inability to prognath, and significantly limited mouth opening and neck mobility (Fig. 1), awake nasal fiberoptic intubation was performed for anesthesia induction and succeeded with difficulty at the third attempt. General anesthesia was then maintained with intravenous propofol and remifentanil to facilitate intraoperative neurophysiologic monitoring. An arterial line was placed. Due to the severe deformity of neck, chest and hip, there was no ideal access to either internal jugular or femoral vein and central venous catheter was decided not to be placed. Instead, three large bore peripheral IVs were initiated. The girl was then positioned prone with both longitudinal and transvers bolsters for chest and pelvis support, with hips and knees flexed. There were no significant changes in hemodynamics or oxygen saturation at this point (blood pressure 100–110/60-65mmHg, SpO_2_ 100%, PaO_2_ 191mmHg at FiO_2_ 50%).

**Fig. 2:**
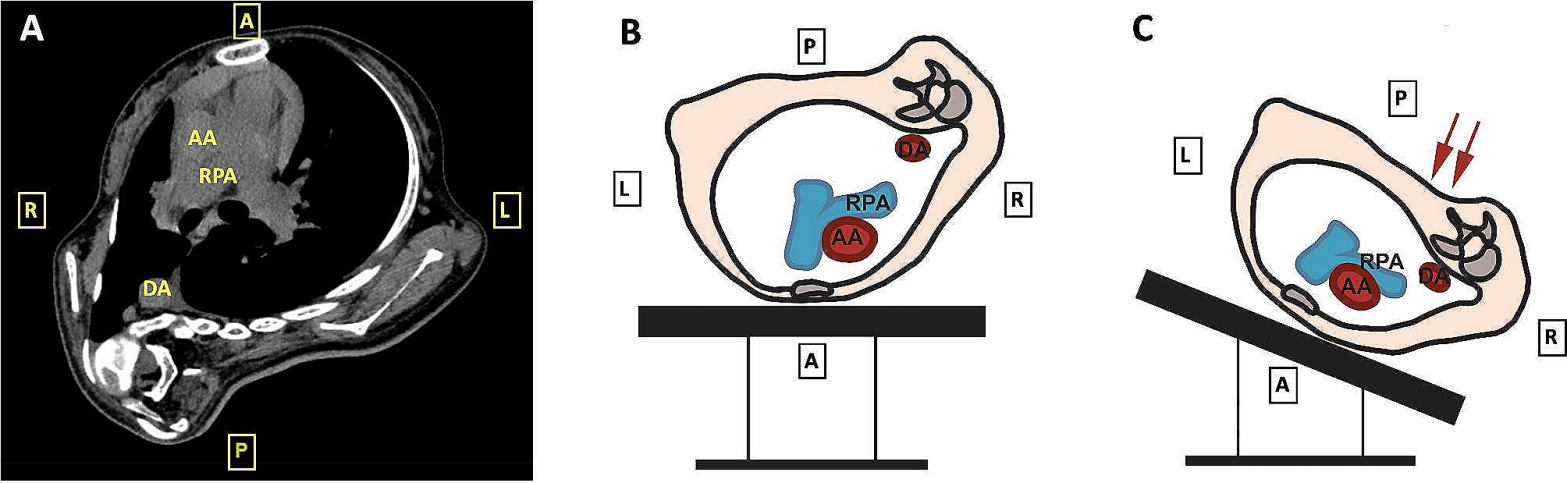
Preoperative CT and schematic diagram of the intraoperative patient positioning. (**A**) CT chest imaging showing a rotated and distorted thoracic cavity with much reduced volume of the right side. (**B**) Patient at the leveled prone position. (**C**) When the surgical table was tilted to the right for periosteal exposure on the left side of the spine, pressure was directly put on the shortest diameter of the significantly deformed thoracic cavity, causing severe compression of the pulmonary artery. CT, computed tomography; AA, ascending aorta; DA, descending aorta; RPA, right pulmonary artery

Surgery started with a midline incision in the back from T2 to L5 and erector spinae muscles were separated. The severe scoliosis formed a rigid right thoracic curve, and the operation table was tilted about 45 degrees to the right to facilitate subperiosteal exposure of the left side of T4-T8. About 2 min after surgical table adjusting, the patient developed significant hypotension (blood pressure 70/40mmHg), tachycardia (heart rate 115 per minute), hypoxemia (SpO_2_ 80% at FiO_2_ 100%) and hypocapnia (EtCO_2_ dropped to 30mmHg) with no obvious change of airway pressure. Breath sounds were clear and symmetrical bilaterally on auscultation. Body temperature was normal, and there was no flushing or rash. At this point, the patient had a total fluid infusion of 500mL crystalloid with estimated blood less than 50mL. The girl was managed with vasoactive medications (repeated ephedrine and phenylephrine boluses and norepinephrine infusion up to a maximum rate of 0.5 µg·kg^− 1^·min^− 1^) but responded poorly in the following 10 min. The surgeons were asked to temporarily stop the procedure, but hypotension and hypoxemia were still refractory. Mechanical compression related with surgical positioning was highly suspected at this point and her preoperative CT was referred. It was noticed that the thoracic cavity was severely distorted and the volume of the right side of the thorax was much reduced. A reasonable explanation was when the surgeons performed periosteal exposure on the left side of T4-T8 with the surgical table tilted right, pressure was directly put on the shortest diameter of the significantly deformed thoracic cavity, causing severe compression of the pulmonary artery, resulting in profound hypoxemia as well as hypotension (Fig. 2). The operation table was then leveled back to horizontal.

Hemodynamics and pulse oximetry started to improve since then, though blood gas showed mild acidosis with still unsatisfied oxygenation index at this point (pH 7.259, base excess − 4.2, PaCO_2_ 52.7mmHg, PaO_2_ 148 mmHg at FiO_2_ 100%, oxygenation index 148). The patient was watched for another 10 min and was stable with a norepinephrine infusion of 0.2 µg·kg^− 1^·min^− 1^ to maintain blood pressure 110/70mmHg and normal SpO_2_ with FiO_2_ 70% at the leveled prone position. After discussing with the surgical team, we decided to continue with the current position, and to complete the surgery with as little invasive approach as possible. Single rod instrumentation was performed on the concave side with spinal fusion from T2 to L5. The surgery went on uneventfully and finished as planned in the next two hours, with a total blood loss of 300mL. Ventilation was noticed to be improved with a lower peak airway pressure compared with the beginning of the surgery (from 32cmH_2_O to 26cmH_2_O) after scoliosis correction. Norepinephrine infusion was reduced to 0.1 µg·kg^− 1^·min^− 1^ and the blood gas was very much improved at the end of surgery (pH 7.366, base excess − 1.7, PaCO_2_ 41.1mmHg, PaO_2_ 409 mmHg at FiO_2_ 70%). The patient was sent to ICU for further monitoring and was successfully extubated on POD1. She did well for the postoperative period and was discharged without complications.

## Discussion and conclusions

Prone position during surgery is known to be associated with physiologic cardiovascular changes including reduced stroke volume, cardiac index, raised central venous pressure, reduced venous return and ventricular compliance [5, 6]. More severe hemodynamic changes may occur in scoliosis children with thoracic deformity and increased chest wall compliance when positioned prone [4, 7]. Though cases of severe hypotension associated with prone positioning have been reported in children with pectus excavatum or idiopathic scoliosis undergoing spine surgery previously, the case we present is unique for several reasons.

To our knowledge, this is the first case to report not only intraoperative hemodynamic instability during prone position, but also accompanied with profound hypoxemia, which has not been described before. Previous reports have discussed severe hypotension in prone position with various transesophageal echocardiography (TEE) findings. Abcejo and colleagues reported a scoliotic child suffered refractory hypotension immediately after prone positioning on the Jackson table and resolved after placed supine [4]. TEE showed a very narrow left atrium in the anteroposterior dimension vulnerable to obstruction, and hemodynamic stability was achieved through moving chest supporting pads to the subcostal region. Bafus et al. and Galas et al. presented severe hypotension in cases of pectus excavatum immediately after prone positioning with TEE showing right ventricular inflow obstruction [8, 9]. Neira et al. reported severe cardiovascular collapse during scoliosis surgery while TEE confirmed right ventricular outflow tract obstruction caused by transverse bolsters [10]. In our case, profound hypoxemia in addition to hypotension made the situation more complicated. For differential diagnosis, airway reasons were first ruled out as the position of the endotracheal tube had been confirmed by fiberoptic bronchoscopy in the first place and there was no apparent change in airway pressure with both lungs clear on auscultation when SpO_2_ dropped. There were also no signs of massive bleeding or anaphylactic shock. Considering the girl developed persistent hypoxemia and hypotension simultaneously, which was very similar as the pathophysiology changes of pulmonary vascular obstruction, mechanical compression of the pulmonary artery was highly suspected. However, it was very difficult to prove this theory with direct evidence due to the limitation of monitoring in this case. It was unpractical to perform TEE examination for this girl due to her very limited mouth opening for less than one finger, apparently not enough to insert the TEE probe. There was even no central line catheter for a central venous pressure. Nevertheless, the preoperative CT gave us a clue and helped with adjusting the surgical positioning to stabilize the patient.

Another point we would like to address is the clinical decision in this case. In most of the previously reported cases of severe intraoperative hypotension at prone position, the surgery was canceled, and the patient was then placed supine for resuscitation and received TEE examination [4, 8–11]. For us, it was also very difficult when neither the SpO_2_ nor the blood pressure responded to active treatment. We had to decide immediately whether to cancel the surgery and put her back to supine to resuscitate, which we were reluctant to do as the general condition of this SMA girl may further deteriorate without surgical correction and internal fixation of the severely deformed spine. Also, the opportunity for her spine surgery was precious. We had made every effort to optimize her preoperative status, achieving possibly the best preoperative status she could get. If the operation were cancelled at this point, the girl may never have a second chance to receive surgery, especially coupled with the extreme difficult airway she had experienced this time. Therefore, we had to quickly find the cause for the pathophysiological changes and reverse this unfavorable situation promptly. Though advanced monitoring techniques like TEE was unavailable in this case, thankfully her preoperative CT helped with timely diagnosis, giving us the opportunity to continue the surgery. As the patient had just experienced a severe episode of hypoxemia and hypotension and was still very fragile, we need to minimize surgical time and reduce blood loss to lower the risk. Considering she had very low body weight and very limited scope of activities caused by SMA, a relatively simple internal fixation would be enough to support her. After fully weighing the risks and benefits, we decided to complete the surgery in the least invasive way and single rod instrumentation was performed. Intraoperative neuromonitoring signals were normal all the time through the procedure, and wake-up test was not performed to avoid further hemodynamic fluctuation. She recovered well from the surgery and has been followed up on 3, 6 and 11 months postoperatively as of the time of writing. Currently she is presented with not only satisfied cosmetic improvement, but also significantly improved sitting ability.

As a rare autosomal recessive neuromuscular disorder, SMA leads to progressive degeneration of anterior spinal motor neurons that cause significant muscle weakness [12]. Rather than for cosmetic purposes, the main purpose for surgical spinal fusion for SMA patients is to prevent scoliosis progression, thereby improve sitting balance and ventilation for better quality of life, which is critical for them [13]. However, perioperative management for SMA patients undergoing spinal deformity surgery is far more challenging than that for scoliosis of other types, for example, idiopathic scoliosis [14]. Anesthesia for these group of patients are known for a particularly high risk of difficult airway and respiratory complications due to restrictive lung function caused by both muscle weakness and thoracic deformity [15, 16]. Conversely, perioperative cardiovascular complications are not common, and there is no previous report considering the hemodynamic instability during spinal deformity surgery in SMA patients [14, 15]. Here we describe a case of successful treatment of intraoperative hypotension and hypoxemia in lack of advanced monitoring due to severe anatomical abnormality and resulted in a positive outcome for an SMA patient who did not tolerate the tilted-prone position, pointing out another major pathophysiologic abnormality that may occur during scoliosis correction surgery.

There are some limitations in this case. Even as experienced anesthesiologists and surgeons, we have not foreseen the profound clinical deterioration preoperatively. We have not recognized position related problem in the first place when the patient was unstable until the preoperative images were referred to. To improve future practice, there are several points learned from this case that we would like to address. In the first place, a rapid, stepwise assessment of critical patients with intraoperative instability is needed to prevent or mitigate complications. A checklist can be helpful for efficient recognition of potential causes and decision making. For spine deformity surgery, checklists have been well established for intraoperative neuromonitoring changes to optimize response [17]. However, major intraoperative issues other than neuromonitoring changes, like the hemodynamic and oxygenation fluctuations we encountered in this case, also require urgent response in patients with severe scoliosis. A modified checklist more extensively indicated for all intraoperative major events, with checking surgical position and preoperative imaging included, would be a useful tool as a reminder for making full use of all available resources, benefitting the rapid diagnosis and appropriate treatment in case of profound clinical deviations.

For patients with severe chest wall deformity, like kyphoscoliosis SMA children with asymmetric and even distorted thoracic cavity, dramatic hemodynamic and oxygen saturation changes should be alerted not only when positioning prone, but also when tilting the operation table intraoperatively which may lead to further compression. According to previous reports, TEE can be useful for timely differential diagnosis in case of intraoperative hemodynamic instability, and could be considered to guide the positioning of the bolsters to minimize external compression during prone positioning when possible, though not practical in our case [10, 11]. Further studies are needed to determine whether hemodynamic and respiratory compromise can be predicted in certain SMA patients prior to surgery, especially those with severe thoracic and spine deformity.

## Data Availability

No datasets were generated or analysed during the current study.

## Abbreviations

SMA: Spinal muscular atrophy
TEE: Transesophageal echocardiography
CT: Computed tomography
